# Optical Optimization of Tandem Solar Cells: A Systematic Review for Enhanced Power Conversion

**DOI:** 10.3390/nano13232985

**Published:** 2023-11-21

**Authors:** Ayesha Razi, Amna Safdar, Rabia Irfan

**Affiliations:** 1 School of Chemical and Materials Engineering (SCME), National University of Sciences and Technology, Sector H-12, Islamabad 44000, Pakistan; 2 School of Electrical Engineering and Computer Science (SEECS), National University of Sciences and Technology, Sector H-12, Islamabad 44000, Pakistan

**Keywords:** optical design, nanomaterial optimization, tandem solar cells, deep learning, power conversion efficiency

## Abstract

Tandem solar cells (TSCs) perform a better adaptation of the incident photons in different-energy-level bandgap materials, and overcome the Shockley–Queisser limit, but they require advanced control over the management of light for optimum performance. Nanomaterials and nanostructures offer a vastly improved control over the management of light. Through different optimization techniques, researchers can gain valuable insights regarding the optimization of various parameters of nano-optical designs. Over the past years, the number of studies on this topic has been continuously increasing. The present study reviews various current state-of-the-art optical designs, and provides an overview of the optimization techniques and numerical modeling of TSCs. This paper collected and analyzed different studies published within the years 2015–2022, using systematic literature review techniques, such as specific protocol screening and a search strategy. Seven different optical designs were extracted, along with their advanced local and global optimization methods, which offer a solution to the optical limitations of TSCs.

## 1. Introduction

Over the past two decades, solar cell technology has progressed at a rapid rate, from supporting niche uses on a tiny scale, to becoming a mainstream source of energy. A dominating fraction of the solar market share is silicon single-junction cells (SJCs), with a power conversion efficiency (PCE) of up to 26.7% [1]. The SJC is limited by the second law of thermodynamics, indirect bandgap, the Shockley–Queisser limit, and a low power yield [2]. Only photons with a higher energy than the SJC’s bandgap are absorbed, which are not fully converted to electrical energy, due to recombination losses. 

Research on multi-junction solar cells (MJCs) emerged, intending to replace SJCs, in the 1980s. The TSC, a classification of MJCs, is viewed as the next phase in solar cell development. The high- and low-energy photons are taken up by the wide-bandgap (WBG) and low-bandgap (LBG) active layers, respectively, hence outperforming the Shockley–Queisser limit. Furthermore, TSCs are also cost-effective, as silicon can act as a substrate material. However, there are some major challenges in the optical design of TSCs, as they require advanced control over the management of light.

Nano-optical materials manage light through a process that involves the use of multiple nanostructures with dimensions in the range of optical wavelength, which can be employed for light trapping [3,4,5,6,7,8,9,10,11], anti-reflection coatings (ARCs) [11,12,13,14,15], and spectral modification materials [16,17]. Through optical simulations, researchers can gain valuable information on various parameters of nanostructures. The research in nanostructure design for TSCs is in its early stages and many studies have been published recently. There has been a lack of effort to thoroughly review the literature regarding the application of pre-existing studies on the theoretical optical design for TSCs. Therefore, the studies must be gathered, to make informed decisions regarding their use.

This study uses the systematic literature review (SLR) technique to explore the area covered by the optical design of nanostructures for TSCs, and provide a comprehensive overview of the current state-of-the-art designs, and current optimization techniques and their computational research. The study also describes, to date, the benefits of designs, and challenges reported by different practitioners. The research questions are as follows:

1. RQ1. What are the current optical designs used in different TSCs, and what are their potential benefits?
2. RQ2. What are the different techniques used for searching for an optimal optical design for TSCs in the literature?
3. RQ3. What are the current learning-based methods for the optical design of TSCs?
4. RQ4. What are the key challenges faced by different optical designs for TSCs?

The protocol of SLR guides the conduct of the research, and allows us to document a list of studies that are pertinent to the specific research questions. The paper is organized as follows: Section 2 comprises the different types of TSC, depending upon the terminal contacts. Section 3 consists of the background of nano-photonics in solar cells and their optimization. Section 4 and Section 5 consist of the SLR methodology steps and results, respectively. Section 6 consists of the extracted data that were able to answer the research questions, with the help of the 124 studies.

## 2. Types of TSC

**Figure 1:**
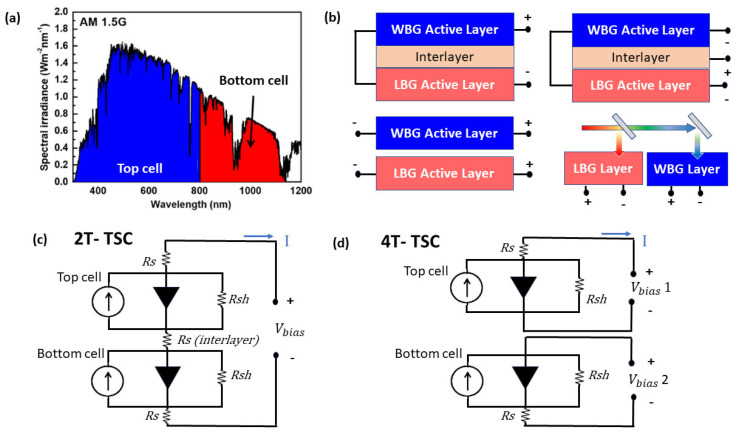
(**a**) Spectral response of the WBG top cell and LBG bottom cell in a TSC. (**b**) Schematic for the basic TSC configuration of a 2T, 3T, 4T, and spectrum-splitting 4T TSC. (**c**) Circuit diagram of a 2T TSC connected in series flowing in forward bias (the blue arrow shows direction of current flow), where R_s_ is the resistance in the cell, R_sh_ represents the shunt resistance, I is the current, and V_bias_ is the bias voltage. (**d**) Circuit diagram of a 2T TSC connected in parallel.

TSCs consists of multiple layers of different, tunable band gap materials that can harvest a broader portion of sunlight, by utilizing different sub-cells that are optimized for specific parts of the spectrum. The best combination for highly efficient double-junction TSCs is top cells with a wider bandgap of 1.7–1.85 eV, and rear cells with a bandgap of 1.12 eV [18]. The high- and low-energy photons are taken up by the WBG and LBG active layers, respectively, as shown in Figure 1a. In SJCs, photons with higher energy than the semi-conductor bandgap are absorbed according to the Shockey–Quessier Limit, and are not fully converted to electrical energy, due to recombination losses. The combination of WBG and LBG semiconductors broadens the absorption range of the TSC from 400–1200 nm. The high absorption of both shorter and longer wavelength photons yields a high current density, ultimately leading to a high PCE. The specific composition and design of the sub-cells can vary, depending on the desired performance and application of the TSC.

Light-trapping and -managing layers, such as window layers, ARCs, or interlayers, may be incorporated, to enhance light absorption, reduce losses, or facilitate charge carrier transportation within the sub-cell. The thickness of each layer in a sub-cell has a significant influence on the TSC performance; therefore, the thickness and material properties also need to be optimized, to avoid current mismatching [19]. TSCs are further divided into categories based on the terminal contacts:

- Monolithic two-terminal (2T) TSC
- Four-terminal (4T) TSC

### 2.1. Two-Terminal (2T)-TSC

The WBG and LBG sub-cells in the 2T TSC are coupled in series circuits, as shown in Figure 1b,c. The WBG sub-cell is synthesized on the LBG sub-cell, with an intermediate recombination junction or interlayer independently, to maximize the PCE. In this configuration, the high- and low-energy photons are taken up by the WBG and LBG active layers, respectively [20]. The fabrication of this device is easy and low-cost compared to other categories. However, current mismatching is high in these TSCs, as the sub-cells are connected in the series circuit. The 2T TSC is further divided into two types, monolithic and mechanically stacked TSCs, depending upon the fabrication method and optical configuration. 

A monolithic TSC integrates multiple sub-cells within a single, continuous device structure. These sub-cells are connected electrically in series, allowing for the efficient cascading of the photo-generated currents. The key advantage of monolithic TSCs lies in the elimination of the need for separate growth and processing steps for each sub-cell, simplifying the fabrication process, and reducing production costs, compared to the mechanically stacked configuration [21]. The architecture enables precise control over the bandgap of each sub-cell, optimizing their absorption characteristics for different parts of the solar spectrum. 

By leveraging materials with varying tunable bandgap materials, monolithic TSCs can achieve an overall high PCE compared to SJCs. However, the first drawback of monolithically produced TSC is the mismatch between the material’s lattice parameters and thermal expansion coefficient, which degrades the device performance. Secondly, to prevent damage to the bottom layers, the top sub-cell needs to be designed via intricate fabrication methods. Additionally, it can be challenging to fabricate interlayers between two conductive sub-cells. A mechanically stacked 2T TSC configuration is also possible, in which individual sub-cells are fabricated separately, and then mechanically stacked on top of each other. The use of mechanically stacked TSCs eliminates the challenges associated with the epitaxial growth and lattice-matching requirements often encountered in monolithic TSCs [21]. However, precise alignment, and effective management of light transmission between the sub-cells, are essential to achieving an optimal device performance.

In recent years, many studies have been conducted on diverse types of TSC, based on contacts, configuration, and materials, specifically focusing on the III–V TSCs utilized in space applications [21,22], Si TSCs [23,24,25,26], gallium-based TSCs [27,28], organic TSCs [29,30,31,32], and emerging perovskite/Si TSCs [33,34,35,36,37]. The summary of the highest recorded PCE from the year 2015–2022 is compiled in Table 1. The gallium-based TSCs are pioneering modules that were designed for space applications. In 2015, FhG-ISE [38] reported a PCE of 30.2% in a 4 cm^2^ 2T containing a three-junction TSC with a wafer-bonded GaInP/GaAs/Si configuration. The GaInP/GaAs sub-cells were not assembled on the Si substrate in this study. Instead, they were produced in an inverted metamorphic configuration on a GaAs substrate, and then moved to the Si bottom cell, using the wafer bonding technique. This method enabled the incorporation of several materials with optimized bandgaps, while guaranteeing an effective charge transfer over the whole cell structure. 

In 2014, Alta Devices revealed a flexible GaInP/GaAs TSC, reaching a PCE of 31.2%, validated by the National Renewable Energy Laboratory (NREL) in 2016 [39]. The authors explore novel device engineering strategies by using thin films as sub-cells, incorporating interlayers to mitigate the losses associated with charge carrier recombination and optical absorption. These additional layers play a crucial role in improving the charge transport properties, and reducing the impact of defects, ultimately leading to a higher PCE. The GaAs are expensive, and have less tolerance to the processing steps for monolithic configurations to be used as a substrate.

**Table 1:** Summary of the record PCE for 2T TSCs from 2015 to 2022 (validated by NREL [40]).

Year	2T-Monolithic TSC Type	Group(s)	PCE (%)	Area (cm^2^)	V_OC_ (V)	Jsc (mA/cm^2^)	FF (%)	Ref.
2015	GaInP/GaInAs	Fraunhofer ISE	30.2	4	-	-	-	[38]
2016	GaInP/GaAs	Alta Devices	31.6	0.999	2.538	14.18	87.7	[39]
2016	Perovskite/Si	Stanford/ASU	23.6	0.99	1.651	18.09	79	[38]
2017	GaInP/GaAs	LG Electronics	32.8	1	2.568	14.56	87.7	[41]
2017	GaInP/GaAs	NREL	32.6	0.248	2.024	19.51	82.5	[40]
2017	GaInAsP/GaInAs	NREL	35.5	0.10031	-	-	-	[40]
2018	Perovskite/Si	EPFL	25.2	1.419	1.787	19.53	72.3	[42]
2018	Perovskite/Si	Oxford PV/Oxford/HZB	25.2	1.088	1.793	19.02	73.8	[40]
2018	Perovskite/Si	Oxford PV	27.3	1.09	1.813	19.99	75.4	[40]
2018	Organic TSC	UCLA	11.5	0.06	1.56	10.07	-	[40]
2018	Perovskite/CIGS	UCLA	22.4	0.042	1.774	17.3	73.1	[40]
2018	Perovskite/Si	Oxford PV	28	1.03	1.802	19.75	78.7	[43]
2019	Organic TSC	SCUT/eFlexPV	13.2	0.041	1.61	11.22	73	[40]
2019	Perovskite/CIGS	HZB	23.3	1.035	1.683	19.17	72.1	[40]
2020	Perovskite/CIGS	HZB	24.2	1.045	1.768	19.24	72.9	[40]
2020	Perovskite/Si	HZB	29.15	1.06	1.897	19.75	77.8	[44]
2020	GaInP/GaAs	NREL	32.9	0.25	2.5	15.36	85.7	[40]
2020	Perovskite/Si	Oxford PV	29.5	1.121	1.884	20.26	77.3	[40]
2021	Perovskite/Si	HZB	29.8	1.016	1.8	-	-	[40]
2022	Perovskite/Si	EPFL/CSEM	31.3	1.1677	1.9131	20.473	79.8	[40]
2022	Perovskite/Si	HZB	32.5	1.014	1.9798	20.24	81.2	[40]

Perovskite, known for its stability and tolerance to damage during the processing and fabrication of TSC devices, has sparked considerable interest among research scientists in recent years. This interest was particularly motivated by the potential of perovskite/Si TSCs with an ideal band gap combination. In 2014, a significant milestone was achieved with the first reported perovskite/Si TSC, which demonstrated an overall PCE of 13.4%, with the top sub-cell and the bottom sub-cell achieving a 6.2% and 7.2% PCE, respectively. The authors further estimated that, with both optical optimizations, a remarkable PCE of 31.6% could be achieved [45]. This groundbreaking result showcased the immense potential of perovskite/Si TSCs, and served as a starting point for further advancements in optimizing their performance. The first perovskite/Si 2T TSC was reported in 2015. The TSC exhibited an overall efficiency of 14.3% [46]. The simulations further highlighted the potential for an even higher PCE, indicating that a PCE of 32% could be attained through the optimization of the transparent electrodes and the interlayers between the sub-cells. 

**Figure 2:**
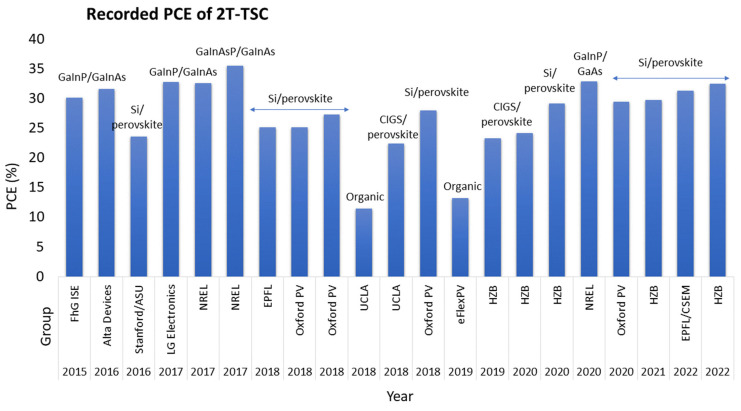
Summary of the highest-recorded PCE of 2T-monolithic TSCs from 2015 to 2022, where FhG ISE stands for Fraunhofer ISE; ASU stands for Arizona State University; UCLA stands for University of California; HZB stands for Helmholtz Zentrum Berlin; EPFL stands for École Polytechnique Fédérale de Lausanne; CSEM stands for Centre Suisse d’Electronique et de Microtechnique; and eFlexPV stands for South China University of Technology.

The PCE of monolithic 2T Si/perovskite increased dramatically in successive years. In 2016, a PCE of 23.6% was recorded, as reported by Stanford University, with a short circuit current density (Jsc) of 18.09 mA/cm^2^ [38]. Three new 2T Si/perovskite structures were fabricated by the Oxford PV group in the year 2018, with PCEs of 25.2%, 27.3%, and 28%, respectively, which exceeded the Si–SJC Shockley–Queisser limit [43]. In 2020, NREL demonstrated the highest-efficiency GaInP/GaAs TSC, reaching a PCE of up to 32.9%. The record of the GaInP/GaAs TSC is claimed to have been broken by the King Abdullah University of Saudia (KAUST) group, through the setting of a record of a 33.2% PCE, currently reported in the NREL chart [40]. Figure 2 shows the summary of the highest-recorded PCE of 2T monolithic TSCs from 2015 to 2022.

### 2.2. Four-Terminal (4T) TSCs

The WBG and LBG sub-cells of 4T TSCs are coupled with optical materials, and electrically separated from one another, which means that individual sub-cells are fabricated separately, and then mechanically stacked on top of each other. Each sub-cell operates independently, absorbing a specific portion of the solar spectrum based on its bandgap, as shown in Figure 1d. In contrast to 2T TSCs, the two sub-cells in this configuration are not dependent on one another; hence, a greater number of electrodes with transparent surfaces are necessary for effectively transmitting light from the WBG top to the LBG bottom cell. 

The greatest drawback of the 4T mechanically stacked configuration is that the electrical top and bottom contacts and electrodes disrupt the transmission of light to the bottom layers. The photons must pass through a series of three translucent contacts with a varying refractive index, which generates a shadowing effect at each terminal contact. The bottom cell yields a low Jsc; hence, the overall performance of the TSC is affected [47,48].

**Figure 3:**
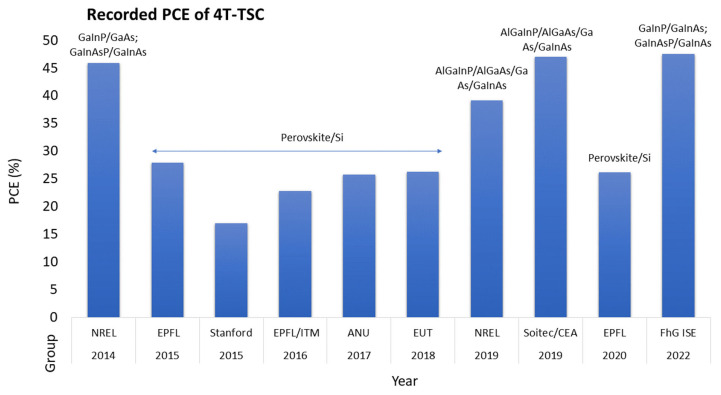
Summary of the record PCEs of 4T TSCs from 2015 to 2022, where FhG ISE stands for Fraunhofer ISE; ANU stands for Australian National University; HZB stands for Helmholtz Zentrum Berlin; EPFL stands for École Polytechnique Fédérale de Lausanne; and EUT stands for Eindhoven University of Technology.

In contrast to 2T TSCs, 4T TSCs can achieve a high PCE over the WBG of 1.5–2.3 eV for the top cell, in combination with a 1.12–1.81 eV bottom cell [49]. Table 2 and Figure 3 show the summary of the highest-recorded PCE of a 4T TSC. In the 4T TSC configuration, the sub-cells are fabricated independently, so there is no need for current matching [50]. However, due to the separate electrical contacts, an insulating interlayer is needed, to avoid optical losses. The overall cost of the 4T device also increases, as more energy-point-tracking sensors are required. Efficient light managing nanostructures can reduce the overall cost of TSCs, by splitting and steering light into the subsequent sub-cell. The 4T TSCs can be divided into two types, depending on the optical design: (I) spectral splitting TSCs, and (II) reflecting TSCs. These optical-designed TSCs are explained in Section 6.1.3 in detail.

In recent years, many studies have been conducted on diverse types of TSC. In 2014, a significant milestone was achieved, with the first reported 4T perovskite/Si TSC, which demonstrated an overall PCE of 13.4%, with the top sub-cell and the bottom sub-cell achieving a 6.2% and 7.2% PCE, respectively. The simulation results estimated that, with both optical optimizations, a remarkable PCE of 31.6% could be achieved [45]. The group employed MoOx coated with ITO to minimize the sputtering damage during the ITO coating to the bottom layers, and to give transverse conductance to the metallization. The next year, nano-material electrodes were optimized for high polycrystalline TSCs. Bailie et al. [51] created a partially translucent device, using transparent silver nanowire electrodes on a perovskite sub-cell. To create solid-state crystalline TSCs, the top sub-cell was physically stacked parallel atop copper indium gallium selenide (CIGS) and crystalline silicon.

Werner et al. [52] produced TSCs at lower temperatures with IZO and MoOx, with high electrical and optical properties and an ideal transparency, in 2015. Over 10% PCE was achieved, with 60% transmission reported in the 800–1200 nm wavelength region to the bottom sub-cell. Through an in-depth analysis of electrical and optical energy losses, Duong et al. [53] established the ideal contact values and general guidelines for the creation of future transparent electrodes and contacts for TSCs. Semitransparent TSCs, with a PCE surpassing 12%, and an increased transmission of 81% in the 800–1200 nm wavelength range, was created via optimizing ITO contacts.

Peng et al. [54] proposed a single-step sol–gel approach for fabricating a high-performance ETL made from indium-doped titanium dioxide (In-TiOx), reaching the PCE of 19% with 77% FF. When designing TSCs of greater areas, Jaysankar et al. [50] addressed difficulties in the transparent electrodes related to sheet resistivity and transparent contact. They built an economical 4T Si/perovskite TSC, using the module-on-cell principle. Finally, in a 4T perovskite/Si mechanically stacked system with the PERL team and IBC cell, a PCE of 26.7% and of 25.2%, respectively, was observed. Zhang et al. [55] also used the same architecture for their TSC. A 25.7% PCE was reached, using a p-i-n planar arrangement top cell, correct optimization of the ITO material characteristics, and light management and control. An increase in the transmission of light in the near-infrared (NIR) region was observed at nearly 90% in the top cell.

The ARC and texturized interface were also utilized in 4T TSC for better light trapping in the active layers. Kanda et al. [56] investigated the optical and optoelectronic properties of the smooth and textured rear end of the bottom cell during the mechanical stacking of 2T and 4T TSC. In the 750–1050 nm wavelength region, they found that the textured interface had a lower reflectance (by 3%) than the flat silicon interface. Furthermore, the average PCE increased from 82% to 88.5%, and the Jsc increased from 13.6 mA/cm^2^ to 14.8 mA/cm^2^. In just over a year, a team from Nanyang Technology University (NTU) and the National University of Singapore (NUS) created a 4T Si/perovskite TSC, with a PCE reaching up to 25.5% [57].

Duong et al. [58] optimized the coating technique and bulk inclusion method for combining two-dimensional and three-dimensional perovskite. A TSC was created via mixing 2D perovskite-based n-butyl ammonia with multiple-cation halide-based 3D perovskite with PERL and an IBC Si-cell band gap of 1.72 eV. The combined PCE achieved with PERL and the IBC Si-cell was recorded as being up to 27.7% and 26.2%, respectively, for a 1 cm^2^ unit area. 

Gharibzadeh et al. [59] used passivation strategies to create perovskite heterostructures with a double cation, with a designed bandgap spanning from 1.6 eV to 1.8 eV. When this device was used in a 4T arrangement, with crystalline silicon as the bottom cell, it produced an amazing efficiency of 25.7% in tandem. Yang et al. [60] made a remarkable breakthrough with a 4T Si/perovskite TSC, obtaining an exceptional efficiency of 28.3% throughout the paper review process. This extraordinary accomplishment is the highest ever documented.

**Table 2:** Summary of the record PCE of 4T TSCs from 2015 to 2022.

Year	4T Monolithic TSC Type	PCE (%)	Area (cm^2^)	V_OC_ (V)	Jsc (mA/cm^2^)	FF (%)	Ref.
2014	4T GaInP/GaAs; GaInAsP/GaInAs	46	0.052	-	-	-	[40]
2015	4T Heterojunction Si/perovskite	28	0.25	0.21	14.5	76	[45]
2015	4T Si/perovskite	17	0.39	0.64	28	66	[51]
2016	4T Si/perovskite	22.8	0.25	1.6	34.8	79	[52]
2017	4T Si/perovskite	25.8	0.17	1.7	38.2	80	[53]
2018	4T Si/perovskite	26.3	0.09	1.7	37.1	80	[55]
2019	4T AlGaInP/AlGaAs/GaAs/GaInAs	39.2	0.09	5.55	8.46	83	[40]
2019	4T AlGaInP/AlGaAs/GaAs/GaInAs	47.1	0.09	-	-	-	[40]
2020	4T Si/perovskite	26.2	0.05	1.7	35.3	80.2	[59]
2022	4T GaInP/GaInAs; GaInAsP/GaInAs	47.6	0.04	-	-	-	[40]

## 3. Fundamental Aspects

### 3.1. Nanostructure and Materials

Reflective and parasitic losses through the different interfaces reduce the practical efficiency of TSCs, and cause current mismatching. Nanomaterials offer improved light management, generally, via two main strategies. First is the reduction of surface reflection, using ARCs or textured surfaces [61,62,63,64]. The other strategy is to increase the optical path in a photoactive layer, using resonant or diffractive effects [65,66,67] The usage of metasurfaces has lately become popular in TSCs. The profile of the dispersed light field is tailored by metamaterials, so that it is properly coupled, trapped, and steered in the solar cell, leading to a high absorbance, while maintaining the same level of current in the sub-cell.

### 3.2. Optimization

Every solar cell optimization requires Jsc and PCE calculations [68]. It is fundamental to use simulation tools and algorithms, given the complex tandem structure and high cost of the fabrication procedure. Due to many parameters, most experimental manufacturing techniques use a trial-and-error approach, which is costly and time-consuming. Calculations based on the first principle of theory [69,70], density functional theory [29], and Monte Carlo have been used effectively in the design approach, though excessive time and computation are required for these calculations [71]. An optimization problem is expressed as a minimization problem.

$$\begin{array}{c} Min\;f\left(x\right)\\ g\left(x\right)\leq 0\;and\;h\left(x\right)=0\\ x_{i}^{L}\leq x_{i}\leq x_{i}^{U} \end{array}$$

where *g*(*x*) and *h*(*x*) are the inequality and equality constraints. *x^L^* and *x^U^* are the lower and upper bounds imposed by the problem. To enhance the PCE, the major key factors are the nano-optical design, thickness, number of active/intermediate layers, materials engineering, and configuration. The optimization techniques of these parameters are classified into the three categories of exhaustive search, and local and global optimization techniques, which are further discussed in Section 6.2.

## 4. SLR Methodology

Details about the steps involved in the SLR process are provided in the following subsections.

### 4.1. Search Strategy and Screening

**Figure 4:**
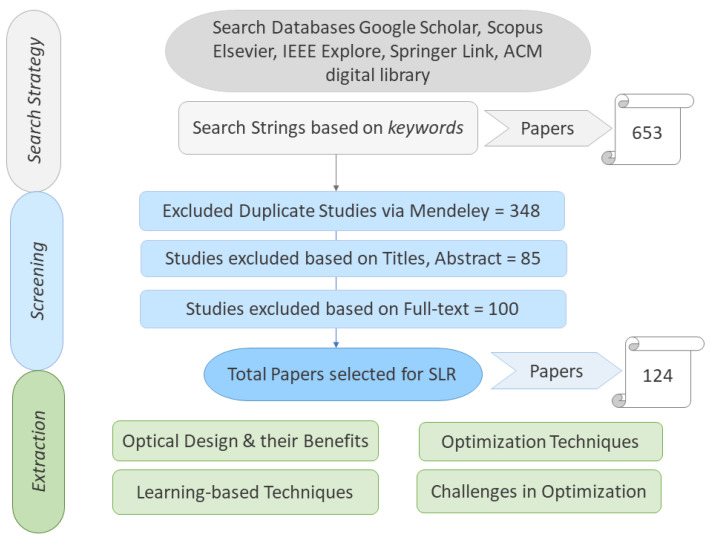
SLR methodology steps classified into three major steps. The first step is searching strategy shown in grey color search is done with three categorical keywords of optical designs with the search databases. The second step is screening by protocol shown in blue. The third step is data extraction in accordance with the research questions.

Database reviews are conducted including Google Scholar, IEEE Explore, Scopus Elsevier, Springer Link, and the ACM digital library. Content from 2015 to 2022, available in the English language, is selected. The advanced search is conducted with three categorical keywords of optical designs (grating, reflectors, metamaterials, nanomaterials), optimization, and TSCs. The abstracts of the collected papers were skimmed first, then the successive sections were analyzed thereafter, resulting in the final list of 124 studies, as shown in Figure 4. The studies were excluded from the database based on duplicate copies via Mendeley. The final selection of papers was carried out after their full text was read thoroughly.

### 4.2. Data Extraction

The data extraction step involves the recording of information that was collected from the various studies regarding the research questions i.e., optical designs, their potential benefits, optimization, current learning-based optimization, and challenges reported by practitioners while optimizing nanomaterials and structures.

## 5. SLR Results

This section aims to provide statistics about the publication sources, citation status, and temporal distribution of the studies.

### 5.1. Publication Source and Citation Overview

**Figure 5:**
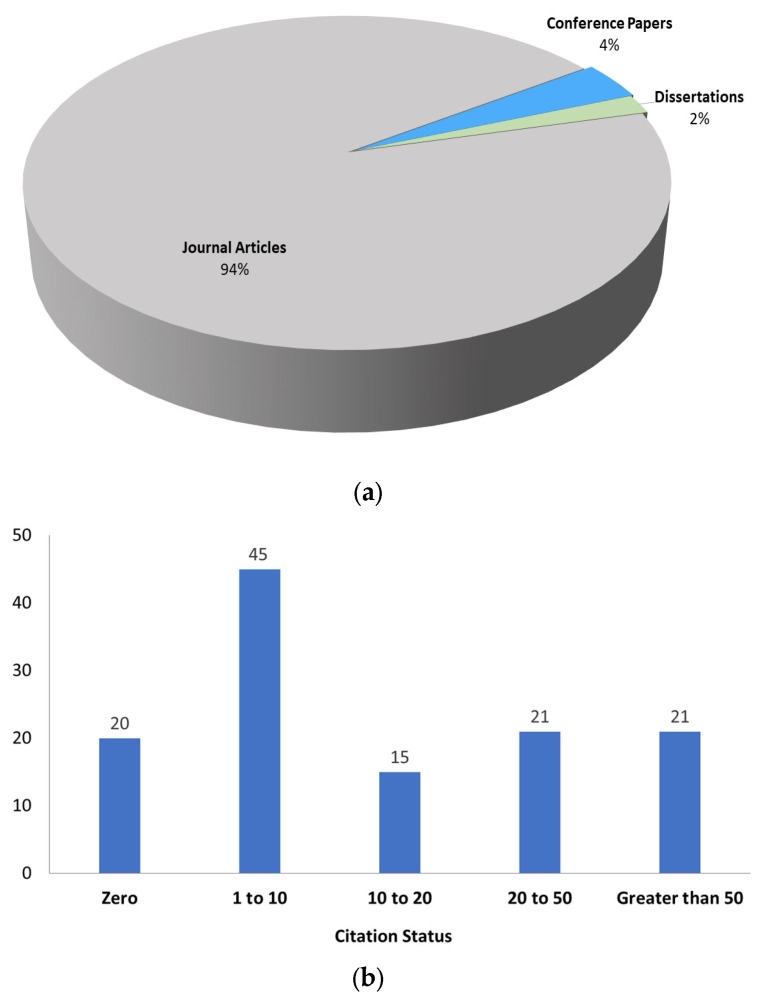
Publication source and citation overview. (**a**) Distribution of studies as per publication source, and (**b**) citation status per number of publications.

According to the most prominent publication sources, Figure 5a,b show the distribution of studies with regard to various publication sources and citation statuses, respectively. The citation rates were collected from Google Scholar. Although the data presented here are not meant to compare the selected studies, they give a rough estimate of the citation rates. Thirty-eight studies have over 50 citations, and have been cited between 20 and 50 times. We expect that the citation rates of the studies will increase significantly.

### 5.2. Temporal Overview

**Figure 6:**
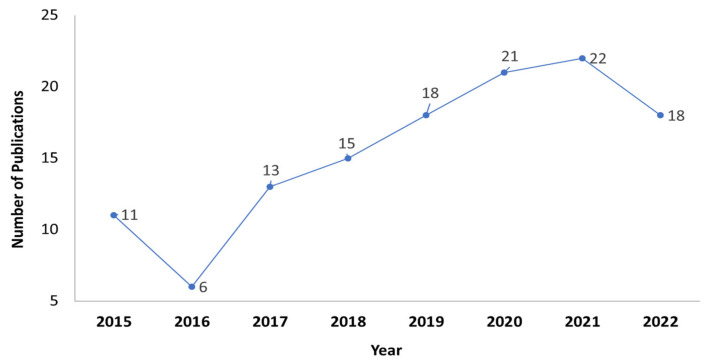
Publications on the optical optimization of TSCs from the year 2015 to 2022.

Figure 6 shows the number of publications on the optimization of TSCs over the years. It shows that the number of publications in this domain has increased significantly since 2016, which reflects the increasing interest in this area in the last 7 years. The number of studies published in the field has increased significantly, and is predicted to increase further.

## 6. Research Question Results

This section consists of extracted data that were able to answer the research questions, with the help of the 124 studies.

### 6.1. What Are the Current Optical Designs Used in Different TSCs, and What Are Their Potential Benefits? (RQ1)

**Figure 7:**
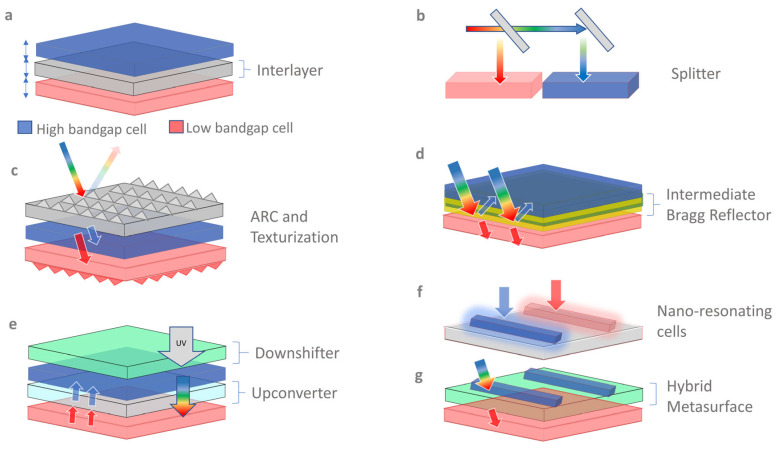
Schematic diagram of TSCs, containing (**a**) an optimized active interlayer; (**b**) splitters; (**c**) a textured bottom cell and an ARC at the front; (**d**) a contrasting indexed Bragg reflector reflecting the longer-wavelength photon WBG cell; (**e**) spectrum-modifying upconverters converting weak photons (shown in red arrows) to high-energy photons (shown in blue colored arrows/, longer wavelengths, and downconverters converting UV light into the visible range; (**f**) nano-resonating cells; (**g**) hybrid metasurfaces formed through embedding WBG cells in nanostructured transport layers for light management.

From the 124 studies, seven different optical designs were extracted, to offer a solution to the optical limitations of TSCs. These designs are classified as (I) an optimized active layer, (II) splitters/selective reflectors, (III) texturized nanomaterials, (IV) spectrum-modifying materials, (V) coupling layers, (VI) hybrid metasurfaces, and (VII) an engineered interlayer for index matching. Figure 7 shows a schematic diagram of a TSC integrated with nano-optical structures. As a result of data extraction, 45% of the studies were found to be related to active layers, spectrum-modifying layers, and metasurfaces.

#### 6.1.1. Active Layer and Thickness Optimization

Optimizing the active layer and thickness in TSCs is a fundamental aspect of achieving a high PCE) and overall device performance. In TSCs, the active layers of both sub-cells must be carefully designed to ensure the complementary absorption of different parts of the solar spectrum. This requires the selection of appropriate materials, with suitable bandgaps and optical properties. Additionally, optimizing the thickness of each active layer is crucial to balancing light absorption and carrier extraction, as excessively thick layers can lead to increased optical losses and carrier recombination, while extremely thin layers may limit light absorption. By carefully adjusting the thickness of the active layers, researchers can tailor the optical and electronic properties, to achieve maximum light absorption and efficient charge extraction, thereby optimizing the PCE of TSCs [3,8,28,31,36,37,72,73,74,75,76,77,78,79,80,81,82,83,84,85,86,87,88,89]. This optimization process often involves a delicate trade-off between optical absorption, carrier transport, and recombination, which necessitates careful characterization and simulation studies, to identify the ideal active layer thicknesses for achieving the optimal device performance. A high PCE can also be yielded with all-perovskite TSCs at low cost. The alloyed perovskite displays a low bandgap (1.12 eV), which can be used in tandem with WBG perovskite material. 

#### 6.1.2. Optimizing Interlayers

Interlayers serve as an important component that facilitates efficient charge transport between the sub-cells in 2T TSCs, enabling the harvesting of a broader range of solar spectra, and maximizing the overall PCE. In the 4T TSC configuration, the sub-cells are fabricated independently, so there is no need for current matching [50]. However, due to the separate electrical contacts, an insulating interlayer is needed, to avoid optical losses. Interlayers facilitate the flow of photogenerated carriers across the device, minimizing losses, and enabling the extraction of higher Jsc [3,4,34,36,65,90,91,92,93]. Additionally, interlayers can help in managing any spectral mismatch or current mismatch between the sub-cells, thereby enhancing the overall performance and stability of the TSC. Between the active layers of the sub-cell, the Fermi energy band level of the HTL (hole-transporting layer) of the top cell and the ETL (electron-transporting layer) of the bottom sub-cell collectively form the interlayer. The layer should match the energy levels of the highest- and lowest-occupied molecular orbital (HOMO) and (LUMO) of the carrier-transporting layers, such that there is minimum resistance and current losses in the interlayer. By optimizing interlayer engineering, the sum of the Voc of the sub-cells is equal to the Voc of the TSC, and the induced current fulfills the proportion of the generation rate of the sub-cell [94]. The choice of materials and engineering for the interlayers is a key aspect of TSC optical design and optimization, as it influences factors such as the charge recombination, optical absorption, and series resistance. The researchers have achieved a PCE of 23.6–30.8% with Jsc 11.3 mA/cm^2^, using materials such as ZnO [92,95,96,97,98,99], ITO [92,96,100,101], FTO [92,102,103], and MoOx [30] as a transparent conducting oxide and interlayer between sub-cells. Furthermore, engineered interlayers can also be used between sub-cells for light management. 

#### 6.1.3. Spectrum Splitter and Reflector Optimization

In a 4T spectral-splitting TSC, planar semi-splitters are designed to split/direct light in a specular direction, as shown in Figure 1b, e.g., a dichroic mirror resulting in high reflection and transmission in the shorter and longer wavelengths in the top and bottom sub-cells, respectively [104]. The benefit of this design is that no additional transparent contacts or electrodes are required. However, the additional cost of the spectral splitter makes this TSC fabrication uneconomical [63]. Through the optimization of the splitter’s design and configuration, the cost can be minimized, as well. Ferhati et al. [7] designed an efficient and low-cost, multilayered, semi-spectrum splitter, using a hybrid metaheuristic approach based on PSO and numerical modeling. The fitness function is comprised of spectral responses. A new class of nano-spectrum splitters has also been introduced recently, which requires no additional optics, and can be easily integrated with all PV modules [105,106,107,108,109,110,111]. 

Another type of 4T TSC is a planar-intermediate reflective module that has contrasting refractive gradients for selective reflection, e.g., Bragg reflectors. The PV mirror is a recently developed idea, in which an inexpensive sub-cell is arranged in a curved sequence, and a dichroic mirror or Bragg reflector is utilized to reflect sunlight toward the more costly sub-cell [112]. This idea has significant manufacturing and installation flexibility, and may be utilized in solar energy collecting technology, such as thermal collectors. 

#### 6.1.4. ARC and Texturization Optimization

To improve the efficiency of the Si-LBG cell, S. Albrecht and colleagues [73] reduced the thickness of the inter-ITO contact. This resulted in a slight increase in the absorption rate and a PCE of up to 17%. The inclusion of selective reflectors [7,104,112,113,114,115,116,117,118,119,120,121], or the texturization of layers can also yield positive benefits, such as increased Jsc [26,37,74,90,100,105,122,123,124]. Light management can also be achieved through reducing the reflected incident light, using an optimized ARC. Various factors, such as the ARC material, the number of layers, the surface texturing, and the layer thickness are being studied to improve the PCE of TSCs. A. Hadi et al. [125] studied single-layered ARCs for GaAs/Si-based TSCs, while X. Guo et al. [12] studied multilayered ARCs, and proposed an easy optimization process, reaching a 27% PCE with 87% absorption. Hassan et al. [13] studied an ARC with a 1D-ternary nano-photonic crystal of silicon oxynitrides (Si OxN) immersed in a silica substrate for 2T TSC with varying thickness targets, which not only increased the absorption in the top active layer, but also achieved the successive transmission of light to the bottom cell in the NIR region (730–1200 nm). The author used the FDTD simulation of 2T TSC architecture, and the TMM method, for the validation of the results.

#### 6.1.5. Metasurface and Metamaterial Optimization

There has been a rising interest in TSCs and metasurfaces working together over the past few years. Metasurfaces have shown potential in improving light absorption, and enhancing the efficiency of solar cells. As per Hossain et al. [9], a metal oxide metasurface is a revolutionary method for increasing a perovskite TSC’s effectiveness. The authors demonstrate that the use of non-resonant metasurfaces can enhance light absorption, and reduce the reflection losses in perovskite solar cells. The metasurfaces are designed using titanium dioxide and silver, which exhibit a high refractive index and a low reflectance, respectively. The proposed meta-surface design is experimentally demonstrated to increase the PCE of the perovskite TSC by up to 25%. Neder [126] presents the design, fabrication, and characterization of 4T perovskite/Si TSCs with an integrated meta-grating spectrum splitter. The meta grating acts as a wavelength-selective filter, allowing greater-intensity photons to enter the Si cell, and reflecting photons with the lower energy that a perovskite cell will absorb. The TSC demonstrated a PCE of 23.6%, which is among the highest-recorded efficiencies for perovskite/SiTSCs. Wang et al. [127] present a dual-layer meta surface with a subwavelength periodic array of gold nano disks on top of a thin HOIP film. The simulation findings conclude that the proposed metasurfaces exhibit a significant enhancement of the absorption of HOIPs, across a broad spectrum of incoming angles and frequencies.

Two contact layers are also required between sub-cells, to collect the charge carriers. Due to the mechanical stacking process, the resulting materials are transparent and high-indexed, to separate the contact layers. Therefore, a thick layer with a low refractive index and a high absorption is needed. The potential benefits of current optical designs are further discussed in detail in Table 3.

### 6.2. What Are Different Techniques Used for Searching for an Optimum Optical Design for TSCs in the Literature? (RQ2)

**Figure 8:**
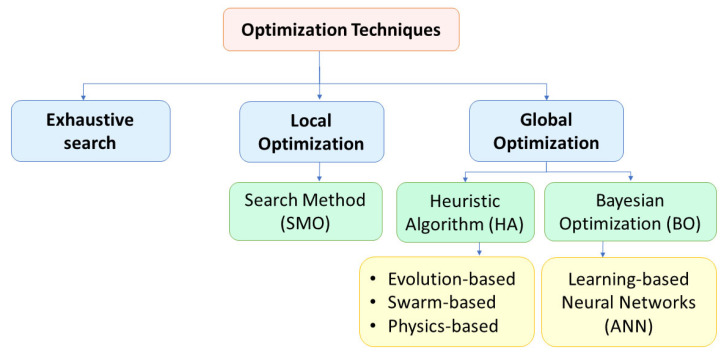
The optimization techniques extracted from the literature.

The optimization techniques for the optical designs (mentioned in Section 6.1) are classified into (I) exhaustive search, (II) local optimization, and (III) global optimization, as shown in Figure 8.

The local optimization method is divided into search method optimization (SMO), while the global optimization has different learning and transfer methods. The heuristic algorithm (HA) is further divided into (I) evolution-based, (II) swarm-based, and (III) physics-based models. 

#### 6.2.1. Exhaustive Search

This technique enumerates all possible permutations of the design variables, and evaluates outputs by finding the global optimum when design vectors are few. As the size and variable of the input vector increase, the computational cost also gets higher. M. H. Elshorbagy et al. [4] mention that an exhaustive search of every layer is carried out separately, due to limitations. Z. Liu et al. [123] also conducted a single-parameter optimization exhaustive search of 4T TSC, to conduct optical analysis, following the Basore model.

#### 6.2.2. Local Optimization (SMO)

This method searches the local optimum without gradient information. Non-linear functions are approximated to near-convex values. SMO can compute and optimize various parameters at the same time. This numerical method can also use the exhaustive search method, searching all possible sets of parameters that are sampled uniformly with each step size. Furthermore, a larger optimization space can be achieved than would be through exhaustive searches, as more samples can be used in the investigation of the material properties and thickness of the active layer, to determine the thermalization losses [8,72,76]. Urs et al. [82] studied organic TSCs by simulating the cell’s optical and electrical features using Setfos, which served as the input for optimization. Both SMO and global optimization techniques were used for a series of various targets. The targets were taken as J–V curves for finding the optimum materials, configuration, and layer thickness of the device. The minima of the generated current were maximized to find the highest PCE. The results yielded a 2% increase in the PCE of the optimized organic TSC configuration.

#### 6.2.3. Heuristic Algorithms (HAs)

These algorithms are advanced randomized search methods that depend on the function value, instead of the gradient and Hessian of the objective. The algorithms can predict the design space, by searching the best-fit input. The genetic algorithm (GA) mimics evolutionary selection, and can handle both unrestrained and restricted optimization problems [164]. It can address optimization problems with continuous, stochastic, nonlinear target functions, and also has the capacity for parallel computing [128]. Manuela [81] compared the exhaustive search, SMO, and GA for the optimization of the Perovskite/Si TSC, and found that the GA finds better solutions than the others. The highest optimized current is 21 mA/cm^2^. However, the GA requires more function evaluation. The differential evolution algorithm (DEA) is well-suited to scouring a huge number of potential solutions. The number of candidate solutions depends on the number of parameters, and the discretization of ranges for optimization [137]. It can solve difficult optimization problems, but it cannot ensure convergence to global minima/maxima [165]. 

Particle swarm optimization (PSO) was motivated by a swarm of birds looking for food. Each bird goes a certain way, until they eventually cluster in a spot, where they obtain food. Potential solutions are referred to as birds, and solutions are found via parameterizing the particulate areas in the search space. Ferhati et al. [7] designed an efficient and low-cost, multilayered, semi-spectrum splitter, using a hybrid metaheuristic approach based on PSO and numerical modeling. The fitness function is comprised of spectral responses. X. Guo et al. [12] optimized the omnidirectional double- and triple-layered ARC via an identical swarm-based algorithm, ant colony optimization (ACO), for practical applications. Furthermore, the refractive index, texturization, coatings, thickness, and number of layers of an ARC can also be optimized for TSCs via this method.

Physics-based models and algorithms are classified as the simulated annealing (SA) algorithm, and the optical transfer matrix method (TMM). The SA is inspired by the annealing of material to increase crystallization and remove flaws. The SA finds a global optimum in a broad search space. If the state space is discrete, then the SA method outperforms other algorithms, because obtaining an estimated global optimum is more essential than finding a precise local optimum, as reported in the literature [31,77]. Few studies are being reported in the literature for the optimization of the junction layer thickness, and determining the electric properties (Jsc, JV curves, and IV curves), as reported by M. Islam et al. [36] and X. Sun et al. [166]. 

On the other hand, TMM is a user-friendly mathematical approach for calculating the plane–wave reflectivity and transmission properties of a substrate of a non/linear material. It uses scalar light scattering theory to calculate the intensity of scattered light modes at textured and rough interfaces. The vector component of the scattered light can also be calculated and modelled via the angular distribution function, using haze variables, as a function of the plane–wave reflectivity and transmission properties. The haze function is the ratio of the scatter light and total specular light [85]. TMM has recently developed, after many years, for bi-anisotropic materials. Substrates that are non-homogeneous in the tangential z-direction can also be handled in the format of a 4 × 4 matrix, using the rigorous coupled wave technique [167]. It can also help in TSC structure optimization by reducing the time and expense of optimizing approaches, based on pure experimentation [91]. TMM can efficiently determine the optimal thickness range of nano-optical structures, i.e., ARCs [13,125,140], the Bragg reflector [128], the interlayer [3,90,91], textured interfaces [85,90,123], and electrodes [124]. Hassan et al. [13] studied the crystal structure of silicon oxynitride (Si OxN) and Bismuth germanium oxide (Bi_4_Ge_3_O_12_) ARCs immersed in a silica substrate for 2T TSC with varying thickness targets, which not only increased the absorption in the top active layer, but also achieved the successive transmission of light to the bottom cell in the NIR region (730–1200 nm). The author used the FDTD simulation of the 2T TSC architecture, and the TMM method, for the validation of the results. As well as the validation and modelling of the optical and electrical parameters, TMM can also predict optimum values. Gulomov et al. [85] studied the optical and electrical properties of varying thicknesses of the top perovskite cell via TMM. TSCs with thickness (800 nm, 80 nm HTL, and 40 nm ETL) yielded Jsc of 21.46 mA/cm^2^. H. Q. Tan et al. [74] also focused on the essential parameters of ETL and HTL, and benefited from the lower computational cost for predicting the optimal design for a bifacial 4T perovskite TSC using ANN trained via BO. The varying thicknesses of the active layers, ETL, HTL, and interfaces was performed via TMM.

Yi et al. [77] reported fast, resource-saving, and accurate solutions through using HA to investigate the structural properties in TSCs under discrete optimization. The study reveals that the SA exceeds both the GA and PSO, achieving the greatest anticipated achievable Jsc. The experimental Jsc and PCE of TSCs reached 15.79 mA/cm^2^ and 23%, respectively; the PCE can be improved to 28%. Wei et al. [31] optimized the thickness of the active layer in polymer TSCs, along with the inner morphologies and other structural parameters, via the combination of different HAs (the SA for inner morphologies, and the GA for determining current properties), and compared them with the SMO. The study discovered that computing all the PCE possibilities takes more than a day in local optimization, while HA achieves the ideal PCE in less than ten minutes.

#### 6.2.4. Learning-Based Optimization

A learning-based optimization is a data-driven approach used to solve complex non-linear optical problems, and predict the design space, through identifying the best input and unidentified regions with previous information. Artificial neural networks (ANNs) learn complicated patterns from large numbers of data. There are several layers in which nodes are linked and aggregated, which are referred to as the hidden nodes, the input data nodes, and the output neurons. The neuron in the input nodes acquires the datasets that are sent into the system, and the neuron in the hidden layers processes it. Yolalmaz and Yuce [111] did an inverse design of nano-spectrum splitters, with the help of ANNs, which saved optimization time compared to search and HAs. However, inverse design poses some significant challenges: (I) phasing plates are required for each frequency, and (II) there are mapping problems. As a data point might relate to several labels, rather than a single class, a mapping issue arises. The mapping problem is addressed in various studies on photonic materials and metasurfaces [156,157,158,159,160]. 

**Figure 9:**
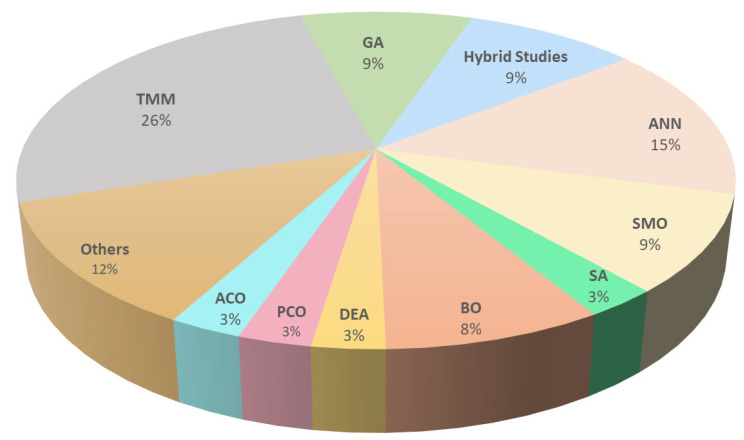
The optimization techniques used in the studies.

Bayesian optimization (BO) uses a Gaussian process, trained using all objective functions, to find new model parameters. It is well reported in the literature that BO is a rapid and highly accurate approach for investigating the Jsc attributes of TSCs. This objective function has the benefit of minimizing reflection and parasitic optical losses, while simultaneously achieving current matching [37]. However, high-dimensional optimization issues are a challenge for BO, because the fundamental problem of maximizing the predicted improvement becomes increasingly difficult. Table 4. shows the optimization techniques used in TSC design. Figure 9 shows the comparative analysis optimization techniques used in different studies. The TMM method was found to be used in the majority of the studies (24%) for the calculation of the optimal thickness range of the nano-optical structures, i.e., the ARCs, Bragg reflectors, interlayer, and textured interfaces. Next came learning-based ANNs, used in 16% of the studies to predict the thickness of the active layers and interfaces, while 10% of the studies used the GA, BO, and hybrid techniques.

### 6.3. What Are the Current Learning-Based Optimization Techniques Used in the Optical Design of TSCs? (RQ3)

The use of learning methods in TSC design problems is a relatively new concept, most of the examples of which have been proposed in the last few years (as shown in Table 5).

One of the greatest optimization problems, the thickness of active layers and interfaces, has been addressed previously via iterative searches [8,37,72,76]. On the other hand, learning-based methods save optimization time compared to the SMO and HA. Several studies [74,77,79] have undertaken the optimization of the thickness of the interface layers and active layers of TSCs.

Tockhorn [84] recently used the global Bayesian algorithm for sensitivity evaluation in 2T monolithic perovskite/Si TSCs, to investigate the effect of the top cell active layer thicknesses on Jsc. The data collection was conducted through a BO, using the finite-element technique (FEM), which solves the non-linear Maxwell equation for anti-reflective texturization and thickness optimization. The group worked on geometrical pyramid textured ARCs, which showed less reflectance (in the range 500–1200 nm) of light through varying the size of the pyramid. By maximizing the minimum of the sub-cell overall Jsc, the PCE of the 2T monolithic TSC was optimized, through assuming ideal light trapping in the bottom cell via Lambertian scattering. The device sensitivity evaluation yielded optimized nano-pyramidal texturized top cells, with a matching Jsc in the top and bottom cells of 20.24 and 20.2 mA cm^−2^. A similar study was carried out by Salah et al. [89], using a systematic BO of ETL, HTL, and the active layer of 2T monolithic perovskite/CIGS TSCs. The findings indicate that an n–p mixed junctions perovskite structure with an electron transporting free material might be a viable replacement for the standard n-i-p structure. As a result of these experiments, the suggested electron-transporting free material perovskite/CIGS cell might be developed, to achieve a PCE of up to 35.36%.

Chaudhary et al. [79] compared the efficiency measures of the Si TSC cell’s output parameters, generated via SCAPS and ANN (trained by the BO). H. Q. Tan et al. [74] also focused on the essential parameters, and benefited from a lower computational cost for predicting the optimal design for a bifacial 4T perovskite TSC, using an ANN trained via BO. C. Yi et al. [77] also reported an ultra-fast, extremely accurate, and computing-resource-saving approach to examining the efficiency of TSCs. The experimental Jsc and PCE of TSCs with the optimum active layer thickness can achieve up to 15.79 mA/cm^2^ and 23%, respectively, and the PCE can be improved to 28%.

### 6.4. What Are the Key Challenges of Optical Designs and Their Optimization? (RQ4)

In semi-reflectors and splitters, a considerable absorption of incoming wavelength is recorded, which induces Jsc degradation in sub-cells. As reported by Ferhati et al. [7], the Jsc decreased from 20 to 17 mA/cm^2^ and 12.4 mA/cm^2^ in the perovskite and CZTS sub-cell, respectively. The low performance of the CZTS sub-cell is attributed to its WBG, due to which it is unable to extend its absorption in the NIR range. Hence, further LBG material is required for absorbing the maximum transmitted light via semi-reflectors and splitters. Furthermore, additional interference layers are also required, with a suitable thickness and refractive index to deal with the coupling effect within the splitter. 

The use of Bragg reflectors has the benefit of reducing the threading dislocation densities, and improving spectral matching. However, their design is computationally intensive, and reflectance in the desired wavelength range requires additional buffer layers with a high refractive contrast, which makes the fabrication and modeling process complex, expensive, and unpractical for fabrication [131]. The most important characteristic of ARCs is the refractive index distribution. Optimization of the vertical distribution of refractive indices is used to optimize the profile of the nanostructures, texture, and fill factor. However, obtaining a high-performance optical structure remains a significant difficulty. It is necessary to perform careful and nuanced optimizations for ARCs, especially for places at high latitudes. When the detailed structural parameters are compared, it is discovered that the lower bound of the indices is more essential, as the difference in indices between the atmosphere and the solar cell is greater, which interface-determines the reflection [12].

The optimizations of gratings and texturization require the computation of boundary-value problems involving structures that are 2D, quasi-2D, or 3D (invariant along one axis, or more axes). When the gratings are periodically simulated in 2D, the processing needs of boundary value issues grow dramatically [127,137]. Multiple design parameters introduced into the optimization process increase the computational load even further. As textured TSC simulation also takes a long time, the amount of free space in the design parameters is also limited. The choice of parameters needs to be made based on the optical performance of the varying layers and thicknesses. Furthermore, extensive search methods need to be employed for shape optimization in geometric nanostructures, which starts at random initial data points, and needs to be trained at each iteration with previous knowledge of functions, to determine the optimum parameter value, until the desired optical properties are obtained. The key difficulty is in creating a customized WBG cell fabrication process that protects the LBG cell’s and recombination layer’s optical properties, while ensuring that the two sub-cells provide a high and equal photovoltage (with the Voc close to the bandgap), and produces high-quality coplanar WBG cells atop double-sided textured LBG cells, to result in better light trapping and a reduced reflection [64]. As reported by Jäger et al. [141], for the optimization of the thickness of the rear-side textured perovskite cell, the thickness of the interface layer was restricted, and the active layer thickness varied, as the texturization of the interface between the WBG cell and LBG cell does not affect the thickness of the front interface between the top active layer and the air. The thickness of the WBG textured active layer is significant for current matching. Some studies also show contrasting results; L. Mazzarella et al. [65] report good optical properties in a textured SiOx interlayer, while K. Jäger et al. [141] report no significant optical response in a textured SiOx interlayer, and light trapping in the top and bottom cell. Double-side textured WBG cells are recommended in papers, due to their anti-reflective effect and light-trapping response.

## 7. Research Gaps

Local optimization and learning-based optimization can generate predictions about the optimum value much faster. They take into account multiple variable parameters at the same time, require a lower computational cost, and lower the risk and cost of trial and error in the fabrication of devices. Advanced learning-based studies can now yield accurate predictions and global minima/maxima regarding the nanoscale structures for better light management, and predict a better design space to enhance the PCE. The active layer can itself be used for light-managing, directing, and restricting layers or metasurfaces. However, there are several opportunities and questions untapped in the realm of optical optimization. These questions have also been raised in past studies. Burgt and Garnett [107] inquire as to whether the use of diffractive structures is better than texturized surfaces for the desired directivity, or vice versa. Another popular question pertains to the overlapping of increased absorption ranges in sub-cells, and the optimization of arrays of nanostructures. These questions require answers, to learn the limitations in the nanostructure designs, and compute a better one. There should also be comparative studies, where parameters including the desired directivity, reflectance/transmission co-efficient, and spectral modification should be studied. 

The development of optimized, efficient, and stable interlayers also remains a challenge. These layers play a crucial role in facilitating charge transport between sub-cells, and ensuring an optimal electrical performance. Further research is needed to identify suitable materials and engineering strategies for interlayers that exhibit excellent electrical conductivity, optical transparency, and long-term stability. Most studies regarding interlayer numerical optimization are exhaustive or parametric-based. Local and global optimization methods can yield better results in 2T TSC, by optimizing interlayers, considering multiple parameters at the same time.

Another research gap lies in the development of novel materials and device architectures to improve the stability and performance of both 2T and 4T TSCs. Despite the significant progress achieved with perovskite materials, their long-term stability under operational conditions is still a concern. The ongoing research efforts should focus on developing more stable perovskite compositions, encapsulation techniques, and interface engineering approaches, to mitigate degradation issues, and enhance device lifetimes.

Efficient light trapping, absorption, and spectral splitting across sub-cells are essential to maximizing the overall device performance. Exploring advanced light management techniques, such as nanostructures, photonic crystals, and anti-reflection coatings, can help improve light absorption, and minimize optical losses, in TSCs.

Lastly, the cost-effectiveness of 2T and 4T TSCs remains a research gap that needs to be addressed. While these devices offer the potential for a higher efficiency, the associated manufacturing costs and scalability challenges need to be overcome to achieve practical implementation. Research efforts should focus on developing cost-effective fabrication methods, scalable deposition techniques, and materials that can be easily integrated into large-scale production processes. The optimization of TSCs can spare the cost served in the trial-and-error approach during experimentation. Recent learning-based studies can reduce the computational cost of optimization, as well. The current study also does not undertake the techno-economical aspect of TSCs. Future studies can compare the cost before and after the optimization of TSCs.

## 8. Conclusions

In this study, an overview of the wide distribution of optical design optimization is performed through conducting an SLR of articles published during 2015–2022. A multi-step process was performed to select the studies, through search strings and a screening process. To afford a descriptive overview of the design, optimization techniques, and status, this study systematically identified relevant studies, resulting in a primary set of 107 articles. The SLR results show that the number of publications has increased significantly since 2016, which reflects the increasing interest in this area in the last 7 years, which is predicted to increase further. Seven different optical designs were extracted from the dataset, which include an active layer, splitters, selective reflectors, gratings, spectrum-modifying layers, coupling layers, hybrid metasurfaces, and interlayers. Most studies were found to be related to active layer optimization, modifying layers, and metasurfaces. Thus, more research efforts are needed regarding hybrid metasurfaces and interlayers. In addition to the optical designs, their optimization techniques were also extracted and classified. Local and learning-based global optimizations are extensively used in the studies. The global HA is further divided into evolution-based, swarm-based, and physics-based models. The TMM method was found to be used in the majority of the studies (24%) for the calculation of the optimal thickness range of nano-optical structures, i.e., the ARCs, Bragg reflectors, interlayer, and textured interfaces. This was followed by learning-based ANNs, which were used in 16% of the studies, to predict the thickness of active layers and interfaces. Learning-based methods have set a new standard, by saving optimization time and computation resources, and they are capable of accurately predicting other wide ranges of properties in highly optimized TSCs.

In conclusion, we have gathered what is already known about optical design and its optimization for TSCs, and how researchers might benefit from them to enhance the PCE. The results can serve as a starting point for researchers, who can then use them to structure and integrate their work, and come up with new optical designs. This study can help practitioners better understand the obstacles they will face while applying optical optimization, and will help them focus their efforts when they do. We created a research protocol before the evaluation, to guarantee that the study selection procedure was as impartial as was feasible. The last notable drawback of this review would be that we did not conduct a hunt for grey literature, because it is notoriously difficult to identify. Overall, this study gives us a perspective on the state of the optical optimization of TSCs at this moment in time.

## Figures and Tables

**Table 3:** Different current optical designs with their potential benefits, extracted from the literature.

Application	Description	Benefits	Papers
Active layer	Tuning of thickness, donor and acceptor doping, material distribution, band gap and inner morphologies.	▪ High lattice and current matching. ▪ High Jsc, improved PCE by 2–25%. ▪ Reduction in parasitic losses.	[3,8,28,31,36,37,72,73,74,75,76,77,78,79,80,81,82,83,84,85,86,87,88,89]
Splitters and semi-reflectors	*Planar-semi* are designed to split/direct light in a specular direction e.g., dichroic mirror.	▪ High reflection and transmission in the shorter and longer wavelengths, respectively.	[7,104,112,113,114,115,116,117,118,119,120,121]
	*Planar-intermediate* have contrasting refractive gradients for selective reflection, e.g., a Bragg reflector	▪ Improved spectral matching. ▪ High conductive layer, to avoid ohmic losses.	[13,22,98,128,129,130,131,132,133,134,135,136]
	*Nano-splitters* of different semi-conductor combinations to split photons.	▪ No requirement for external optics. ▪ Lower bulk recombination rates.	[105,106,107,108,109,110,111]
Texturization and gratings	An *ARC* with a textured interface provides a gradually varying refractive index gradient.	▪ Reduce the amount of reflected incident light, and maximize transmission.	[11,12,13,14,15,27,81,84,101,125,137,138,139,140]
	Selective carriers, electrodes, and contacts.	▪ Reduce the shadowing effect. ▪ Compatible for large-scale fabrication.	[26,37,74,90,100,105,122,123,124]
	*A Lambertian scatterer* scatters weak unabsorbed wave-modes obliquely.	▪ Light trapping. ▪ Reflection losses reduced by 3 mA/cm^2^.	[35,61,123,126,141,142]
	*Geometrical textures* have specific angles, which cause internal refraction.	▪ Causes a coupling effect and light trapping. ▪ High current matching.	[3,9,10,56,64,81,90,141,143,144]
	*Resonant materials* scatter incoming light, and route it to the active layer.	▪ Enhanced light absorption (50–88%). ▪ Affects phase, emission, and amplitude	[16,105,127,137,145,146,147,148,149]
Spectrum modifying layers	*An up-converter* combines many low-energy states into a high-energy state.	▪ Directs light into multiple high modes. ▪ Enhanced near-fields are emitted.	[150]
	*Downshifter* materials convert near-UV light into the visible region.	▪ No need for interlayers, and can act as an ARC. Re-emits visible light with a high yield.	[103,107,151,152,153]
	*Metasurfaces* contain arrays of metamaterials with varying material parameters and periodicity.	▪ Control the light’s phase and amplitude. ▪ Thin, lightweight, easily integrated, low-cost, PCE enhanced by 5–10%.	[107,127,153,154,155,156,157,158,159,160,161]
Coupling layers	Coupling layers change the light intensity in the sub-cells.	▪ Increases the PCE up to 5–8%. ▪ High open-circuit current (~1.12 V).	[91,120,162]
Hybrid meta-surfaces	Hybrid metasurfaces are formed via embedding an active layer in the nanostructured transport layers.	▪ Light trapping and effective transmittance. ▪ Reduced parasitic and reflective losses by 62%, compatible for large-scale fabrication.	[4,5,107]
Interlayers	A refractive-index-matching layer between sub-cells.	▪ Low optical losses in the top cell, or due to light coupling in adjacent layers.	[3,4,13,34,36,65,90,91,92,93,95,99,163]

**Table 4:** Optimization techniques used with regard to the structural properties and functional design in different TSCs.

Parameters	Optimization Technique	Solar Cell	Optical Functional Design	References
Optical design (interlayer, texture, ARC, splitter, metasurface, grating)	TMM	4T-Perovskite–Si	Textured front + interlayer	[90]
	TMM	2T-all Perovskite	Interlayer thickness	[92]
	TMM	4T-Perovskite-cSi TSC	Interlayer thickness	[91]
	TMM	Multilayered TSC	Antireflective metasurfaces	[140]
	TMM	2T-GaAsP-Si TSC	ARC	[125]
	TMM	2T- Perovskite–Si	ARC + interlayer	[13]
	TMM	4T-GaAs-Si TSC	Lambertian scatterer	[123]
	TMM	4T-all Perovskite TSC	Carrier selective electrode	[124]
	BO	2T-Perovskite-Si TSC	ARC, texture, thickness	[84]
	PSO	2T-Perovskite-CZTS TSC	Spectrum splitter	[7]
	GA	2T-Perovskite-Si TSC	Bragg reflector	[128]
	GA	4T-Perovskite-(c-Si) TSC	Geometrical texturization	[144]
	Exhaustive + SMO + HA	2T-Perovskite-Si TSC	Junction layers, ARC, and texture	[81]
	PB	2T-Perovskite-Si TSC	Junction layers	[36]
	BO	Perovskite-Si TSC	Texturization	[141]
	DEA	Thin-film a-Si TSC	Plasmonic grating	[137]
	ANN	All PV modules	Nano-spectrum splitters	[111]
Active layers (bandgap, thickness, donor-acceptor concentration)	SA	2T-Perovskite/Si TSC	Thickness	[37]
	SMO	2T, 3T- TSCs	Thickness, band gap	[76]
	Modified SMO + HA	2T-Perovskite-CIGS, Perovskite-GeTe TSC	Thickness	[72]
	SMO + GA + SA	2T-polymer TSC	Thickness, donor/acceptor concentration, domain size	[31]
	SMO + GA + SA	2T-Perovskite-Si TSC	Thickness	[81]
	SMO + HA	Organic TSC	Thickness, material parameter	[82]
	SMO	Perovskite-Si TSC	Thickness	[8]
	SMO	Perovskite-Si TSC	Thickness	[141]
	SA	2T-Perovskite-Si TSC	Thickness	[73]
	ANN	2T-Perovskite-c-Si TSC	Thickness	[77]
	ANN	Si TSCs	Thickness	[79]
	ANN + GA + TMM	4T-bifacial Perovskite TSC	Thickness, interface, electrodes	[74]
	ANN	Perovskite TSCs	Bandgap	[80]
Material properties	TMM	2T-all Perovskite	Interlayer	[92]
	HA (GA + PSO + SA)	2T-Perovskite-c-Si TSC	Structural parameters	[77]

**Table 5:** Learning-based methods for TSC optimization.

Reference	Method	Simulation	Input	Output	Error (MSE)
[79]	ANN (trained by BO) containing 1 hidden layer and 40 neurons	MATLAB and SCAPS for validation with 3600 training dataset, 1028 validation set	▪ Spectral power density ▪ Temperature ▪ The thickness of the (p-i-n) Si layer	Current density	0.0037
[77]	ANN (15-layered) with 5 hidden layers	FDTD for numerical simulations with 12,500 training datasets	Thickness of: ▪ Glass, ITO ▪ PCBM ▪ Perovskite ▪ PEDOT	Current properties	0.052
[74]	ANN (trained by BO) with 2 hidden layers	TMM and raytracing simulations	Thickness of: ▪ Perovskite ▪ Electrodes	PCE	-
[80]	CNN (100 hidden fully connected neurons), 4 convolutions	Vienna ab initio simulation package for computed structural parameters, containing 862 training dataset	▪ 380 different metal halide perovskite compositions ▪ Crystal structure ▪ Lattice constant	Band gap, lattice constant, and octahedral angle of perovskite	0.02
[141]	BO	SMO (Newton’s method) for thickness	▪ Periods ▪ Aspect ratio ▪ Thickness	Textured interfaces of rear and front sides	-
[84]	BO	FEM for pyramidal textured simulations and thickness optimization	▪ Texture ▪ Thickness of top cell	Current density, Jsc	-

## Data Availability

Data are contained within the article.
